# Clinical assessment of parietal lobe function

**DOI:** 10.1136/pn-2023-003746

**Published:** 2023-06-16

**Authors:** Younes A Tabi, Masud Husain

**Affiliations:** 1 Neurology Department, University Hospital Kiel, Kiel, Germany; 2 Nuffield Dept Clinical Neurosciences, University of Oxford, Oxford, UK

**Keywords:** COGNITION, COGNITIVE NEUROPSYCHOLOGY, NEUROPSYCHOLOGY, CLINICAL NEUROLOGY

## Abstract

The notion of specific assessments of the function of a particular lobe of the brain is in many ways archaic. Advances in our understanding of brain network function have revealed that brain functions are underpinned by large-scale networks with long range connections between cortical distant regions. It would, therefore, be more correct to discuss the contributions of parietal areas to specific functions. Nevertheless, in clinical practice, as we show here, simple bedside assessment can still often point towards parietal dysfunction, or at least reveal an impairment in a function to which parietal regions normally contribute.

## History

The history from the patient or from an informant may give clues to parietal lobe dysfunction. Misjudgement of the spatial location—both direction and depth—of objects, bumping into furniture, knocking over items or spilling drinks should raise suspicions of a visuospatial deficit. Often this is described as clumsiness. If this is the case, it is worth enquiring further by asking for a more detailed description. ‘Clumsiness’ can also refer to difficulty in co-ordinating the hands to open objects such as tins, jars and toothpaste tube tops, or to use cutlery or tools, as might be the case in limb apraxia. It is therefore crucial to understand precisely where in the pathway, from perception to action, the problem might be occurring because a disruption anywhere in this sensorimotor processing stream can lead to significant functional deficits. Sometimes, family members might report that the patient seems to miss seeing things to one side (suggesting unilateral neglect) or even straight in front of them (suggesting the severe attentional restriction of simultanagnosia).

## Rapid bedside examination

Clinicians might believe that the assessment of parietal lobe functions requires a lot of time, arcane tests or even special training. However, useful assessment of parietal lobe function can be performed in even a few minutes when time is limited. We suggest the following:

### Visual inattention

Get the patient to fixate centrally on your nose, raise both your hands, and transiently extend (flick up) either your left or right index finger to assess their response to a single stimulus in either their right or left visual fields. Then test with both left and right index fingers transiently and simultaneously to determine whether there is any visual extinction when there are bilateral stimuli. Extinction refers to consistent awareness of the stimulus only to the left or only to the right, but lack of awareness of both stimuli when presented simultaneously.^1^ It is brought out by using transient presentation of stimuli.

In the tactile domain, one can test for extinction just as one does for visual extinction, but now using transient, light touches to the left or right hand, or both. Thus, patients with right parietal lesions and left tactile extinction may respond to touch on either hand, but report only the touch to the right hand following simultaneous bilateral touches.

### Constructional apraxia

**Figure 1:**
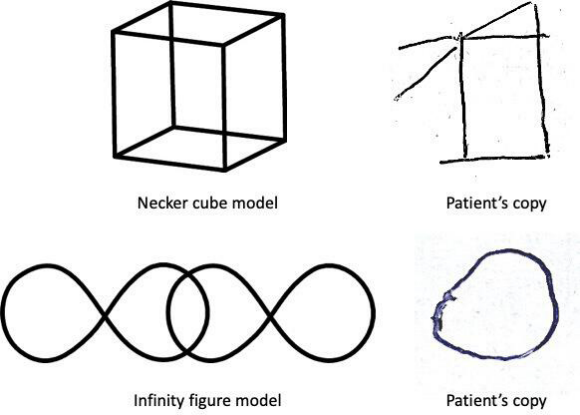
Copying performance. Constructional apraxia or visuoconstructive deficits can be apparent by asking for a copy of the Necker cube or the infinity figure. This patient appears also to show neglect of left-sided elements of both figures in her copies. These model figures are taken from the Addenbrooke’s Cognitive Examination but it is easy for the clinician to draw these at the bedside and ask the patient to copy them.

Draw a Necker cube and an infinity figure on a sheet of paper (figure 1). Ask the patient to make copies. Constructional apraxia, also sometimes referred to as visuoconstructive deficits, can often be apparent with such simple bedside assessment.^2^ Typically, this form of visuospatial deficit has indicated inferior right parietal lobe dysfunction, from various pathologies, ranging from acute-onset strokes to slowly progressive neurodegenerative conditions such as Alzheimer’s disease, dementia with Lewy bodies, corticobasal syndrome or even tumours. An alternative to the Necker cube or infinity figure is to request the patient to draw a clock that shows a particular time. Constructional apraxia can be due to deficits in visuospatial perception, inattention, spatial working memory, deficits in gaze control or combinations of these.

Neglect of elements to one side of a figure may be apparent on such copying tasks, as shown in figure 1. If there is evidence of neglect on copying, it might be useful to perform follow-up tests such as visual cancellation by asking the patient to cross out (or ‘cancel’) all the targets displayed on a sheet of paper.^1^ This is effectively a visual search task. The examiner can make a simple cancellation test by simply drawing a series of randomly oriented lines across the sheet. Adding distractor items (eg, circles) makes the test harder. Cancellation tests can also be downloaded from online resources.

Severe neglect most often follows stroke, particularly right hemisphere stroke, affecting the parietal lobe. Milder forms can be present in more slowly developing conditions. One reason why neglect is so much more apparent in stroke is the acute onset of pathology, which may have sudden, dramatic effects across the network of brain regions connected to the lesioned area. Thus, parietal strokes can have extensive remote effects (diaschisis) on the frontal regions connected to it. Slower pathologies, such as a tumour, may give time for compensatory changes to occur across brain networks and therefore do not commonly lead to severe inattention.^3^

### Limb apraxia

**Figure 2:**
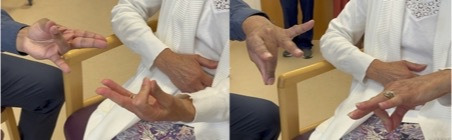
Limb apraxia. Copying meaningless gestures is a simple bedside assessment of left parietal function. Here, the patient (right) is copying the gestures shown by the examiner (left).

While inattention is more severe with right parietal dysfunction, limb apraxia [Bibr R4](of both left and right hands) is most prominent with inferior left parietal pathologies.^4^ ^5^ There are several ways to assess different aspects of praxis but a rapid test is performed by asking the patient to copy meaningless gestures of the hand (figure 2). This is an assessment of what has been termed ideomotor apraxia, the inability to put the hand in the right configuration for an action or simply to copy it in the absence of weakness, sensory loss or other confounding factor. Ideomotor apraxia can also be assessed by asking the patient to pantomime a gesture (eg, waving goodbye or hitching a lift) or use of a tool (eg, use a screwdriver, cut bread, brush their hair). But often assessment of copying a meaningless configuration of the examiner’s hand can be very revealing (figure 2).^5 6^ Ideomotor limb apraxia is observed after left parietal stroke but also in neurodegenerative conditions affecting the left parietal lobe such as Alzheimer’s disease or corticobasal syndrome. It can also occur with left frontal lesions.

The reader might wonder how such a test is ecologically valid: Why is there a be a brain system devoted to copying meaningless gestures? The answer is most likely that human evolution was associated with successful development of a range of tools, each of which requires shaping the hand to use it correctly. The human hand has a remarkable 27 degrees of freedom, permitting a myriad of configurations and a huge repertoire of actions. Learning to use new tools—as well as creating them—requires the ability to transform visual information about hand posture into the motor commands required to achieve that configuration. With 27 degrees of freedom, the permutations are huge and it seems that humans have developed a left hemisphere system devoted to this task.^6^ The right parietal system for visuoconstructional abilities might also be of evolutionary significance, given our abilities to make and copy plans for both small and large scale projects,^6^ essential for the transmission of many human designs, from weapons through to buildings, depictions of landscapes, food sources and maps.

These three tests—of attention, visuoconstructive skills and limb praxis—provide a rapid means to assess left and right parietal function at the bedside, without any special tools or test materials.^7^ More detailed examination can be performed if there are more available resources and time.^8^

## Assessing other aspects of function

### Visual localisation

Visual mislocalisation can be tested first by briefly presenting a dot on an otherwise blank sheet of paper and then presenting a second sheet with numbers at different locations. Patients must report the number that best approximates the dot’s location. It can also be assessed less purely by asking patients to count the number of dots on a sheet of paper. However, impairments on dot counting might also be due to inattention or spatial working memory deficits, both of which can also occur with particularly right parietal dysfunction. A version of dot localisation, without brief visual presentation, is used in cognitive screening tests (eg, Addenbrooke’s cognitive examination) and for extensive neuropsychological batteries today (eg, visual object and space perception battery). Visuospatial working memory can be tested at the bedside by tapping out a sequence of spatial locations and asking the patient to do the same. More formally this can be performed using the Corsi blocks.

### Touch

Parietal lesions can classically lead to a ‘discriminative’ sensory loss. Two-point discrimination, position sense, texture discrimination, stereognosis (ability to identify by touch objects placed in the hand) and graphesthesia (recognition of numbers scratched on the hand) may all be impaired. Usually, this ‘cortical sensory loss’ is more prominent in the arm than the leg and follows damage to the contralateral anterior parietal lobe or its connections.

### Visuomotor control/integration

Unlike astereognosis, tactile agnosia refers to selective impairment of tactile object recognition in the absence of a clinically demonstrable basic sensory impairment. With their eyes closed, patients with tactile agnosia cannot recognise familiar objects placed in the hand contralateral to the lesion, which often involves the inferior parietal lobe or posterior insula.

### Other types of praxis

It is possible to characterise limb praxis deficits further than described in the rapid bedside assessment above.^6^ In addition to examining for ideomotor apraxia, it is possible to assess ideational apraxia, although the use of this term has been extremely confusing. Often it is used to refer to an impairment in the ability to perform a series of actions, for example, asking a patient to make a cup of tea or simply to explain the sequence of acts. They may perform or report each element of the sequence but in an incorrect order. However, some argue that this really represents an inability to recall previously well-established actions such as that for object use—an ‘amnesia of usage’. Others prefer a different term—conceptual apraxia—to specify a defect in the knowledge required to select and use tools and objects.

## Co-occurrence of signs and how they define syndromes

### Attention and Bálint’s syndrome

Simultanagnosia is a severe restriction of attention such that the patient can attend to only one item at a time. In the visual domain, this can be detected by asking the patient to describe a scene from a large photograph, for example, in a magazine, or the famous ‘Boston Cookie Theft’ picture (available on the web). Patients may describe one part of the picture at a time, unable to perceive all elements of the scene simultaneously, or to apprehend the scene in its entirety.^9^ Simultanagnosia occurs with bilateral parietal dysfunction and is one component of the triad of Bálint’s syndrome (see section '‘Visuomotor control and Bálint’s syndrome’). Simultanagnosia is now most commonly observed in degenerative conditions such as posterior cortical atrophy,^10^ which is most often due to underlying Alzheimer’s disease, but it can occur after rare bilateral watershed strokes.

### Visuomotor control and Bálint’s syndrome

Optic ataxia is a second feature of Bálint’s syndrome. This manifests as misreaching to peripherally presented visual targets and can occur with either left or right superior parietal dysfunction.^11^ This sign can be brought out only by getting the patient first to fixate centrally on the examiner’s nose, and reach rapidly to the examiner’s finger presented at different locations in space. Optic ataxia has nothing to do with cerebellar ataxia. Patients with optic ataxia misreach to targets in their peripheral visual field but can accurately reach targets that they are allowed to fixate. They do not show intention tremor of their hand as the movement unfolds that is so characteristic of cerebellar disease.

Gaze apraxia (difficulty in shifting the eyes to peripheral visual targets) is the third and final feature of Bálint’s syndrome. It is attributable to bilateral inattention rather than a primary oculomotor deficit. Patients with gaze apraxia can make saccadic eye movements to command but have difficulty perceiving visual targets and directing their eyes to them. Some clinicians refer to this as ‘sticky fixation’: the eyes seem to be staring straight ahead without responding to visual information in the surrounds.

### Dyscalculia and Gerstmann’s syndrome

Patients with left parietal lesions or neurodegeneration can also show dyscalculia. This is best tested by asking people to perform multidigit addition and subtraction (eg, 294+12 or 171–48). The clinical value of Gerstmann’s syndrome—dyscalculia, dysgraphia, finger agnosia or the inability to distinguish between fingers and left-right disorientation—has been controversial and disputed. It has been associated with left temporoparietal lesions.

### Language and verbal working memory

Although clinicians would often not consider language and verbal working memory to be classical parietal functions, they can nevertheless be sensitive to left parietal damage. Parietal dysfunction in the left hemisphere can, for example, be associated with conduction aphasia. This is characterised by fluent speech but with phonemic errors, and intact comprehension but poor repetition. Classically, this has been framed as a ‘disconnection syndrome’ involving the arcuate fasciculus and leading to disconnection of superior temporal lobe language zones (Wernicke’s) from Broca’s area. Left temporoparietal lesions or neurodegeneration can also lead to verbal short term or working memory deficits. At the bedside, patients may be able to repeat short sentences (‘I went to Cheltenham’) but have difficulty with longer ones (‘I took the train to Edinburgh last week’). This is a characteristic feature of logopenic primary progressive aphasia,^11^ usually due to underlying Alzheimer’s disease.

Further assessment requires deeper cognitive testing, ideally by a neuropsychologist or someone experienced in performing neuropsychological tests.

**Key points**

- Parietal function can be tested rapidly at the bedside with three simple tests, of visual inattention, constructional apraxia (visuosconstructive deficits) and limb apraxia.
- More detailed or follow-up testing might incorporate visual cancellation (for neglect), visual localisation (dot counting), visual working memory (tapping out sequences of locations) and graphesthesia.
- Bilateral parietal dysfunction may be associated with the triad of Bálint’s syndrome: simultanagnosia (attending to only one item at a time), optic ataxia (misreaching to peripheral visual targets) and gaze apraxia (difficulty in making saccades to peripheral visual targets), all readily elicited at the bedside.

**Further reading**

- Hodges JR. *Cognitive Assessment for Clinicians*. 3rd ed. Oxford: : Oxford University Press 2017.
- Husain M. Parietal lobes. In: Husain M, Schott JM, eds. *Oxford Textbook of Cognitive Neurology and Dementia*. Oxford: : Oxford University Press 2016. 51–8.
- Vallar G, Coslett HB, editors. *The Parietal Lobe. Handbook of Clinical Neurology Vol 151*. Elsevier 2018.

## Data Availability

Data sharing not applicable as no datasets generated and/or analysed for this study.
